# Intraovarian injection of platelet-rich plasma in assisted reproduction: too much too soon?

**DOI:** 10.1093/humrep/deab106

**Published:** 2021-05-08

**Authors:** Lloyd Atkinson, Francesca Martin, Roger G Sturmey

**Affiliations:** 1 Centre for Atherothrombosis and Metabolic Disease, Hull York Medical School, University of Hull, Hull, UK; 2 Division of Developmental Biology and Medicine, School of Medical Sciences, Faculty of Biology, Medicine and Health, The University of Manchester, St Mary’s Hospital, Manchester, UK

**Keywords:** ovarian rejuvenation, platelet rich plasma, premature ovarian failure, oogonial stem cells, cytokines

## Abstract

The prospect of ovarian rejuvenation offers the tantalising prospect of treating age-related declines in fertility or in pathological conditions such as premature ovarian failure. The concept of ovarian rejuvenation was invigorated by the indication of the existence of oogonial stem cells (OSCs), which have been shown experimentally to have the ability to differentiate into functional follicles and generate oocytes; however, their clinical potential remains unknown. Furthermore, there is now growing interest in performing ovarian rejuvenation *in situ*. One proposed approach involves injecting the ovary with platelet rich plasma (PRP).

PRP is a component of blood that remains after the *in vitro* removal of red and white blood cells. It contains blood platelets, tiny anucleate cells of the blood, which are responsible for forming athrombus to prevent bleeding. In addition, PRP contains an array of cytokines and growth factors, as well as a number of small molecules.The utility ofPRP has been investigatedin a range of regenerative medicine approaches and has been shown to induce differentiation of a range of cell types, presumably through the action of cytokines.

A handful ofcasereports have described the use of PRP injections into the ovaryin the human, and while these clinical data report promising results, knowledge on the mechanisms and safety of PRP injections into the ovary remain limited.In this article, we summarise some of the physiological detail of platelets and PRP, before reviewing the existing emerging literature in this area. We then propose potential mechanisms by which PRP may be eliciting any effects before reflecting on some considerations for future studies in the area. Importantly, on the basis of our existing knowledge, we suggest that immediate use of PRP in clinical applications is perhaps premature and further fundamental and clinical research on the nature of ovarian insufficiency, as well as the mechanism by which PRP may act on the ovary, is needed to fully understand this promising development.

## Introduction

Female infertility is recognised by the World Health Organisation (WHO) as a global public health issue (Macaluso *et al.*, 2010), with more than one million cycles of IVF being performed globally each year since 2005 (Zegers-Hochschild *et al.*, 2014; Adamson *et al.*, 2016). Female infertility can arise from a range of conditions, including endocrine dysfunction, implantation failure, endometriosis and uterine fibroids, as well as pathologies related directly to the ovary, including polycystic ovary syndrome (PCOS), primary ovarian insufficiency (POI), environmental factors and inflammatory disease. However, ‘ovarian exhaustion’ is a natural part of the ageing process. In the past 50 years, the mean age at which women have their first child in the UK has increased from 23.8 to 30.7 years (Office for National Statistics, 2020), suggesting that women are delaying childbearing. The impact of delayed childbearing means that women are moving closer to the period of climacteric for conception, and, in many cases, women are choosing not to reproduce until much later. One consequence of this has been a rise in fertility treatment and a rise in the age of women attending for medical investigation. Indeed, in the UK alone, the mean age of women attending for IVF treatment has hovered around 35 for the past 20 years (Human Fertilisation and Embryology Authority, 2020). Since the advent of clinical IVF in 1978 (Steptoe and Edwards, 1978) and associated Assisted Reproductive Techniques (ARTs), it has been possible to treat infertility in a number of cases. However, such approaches are reliant on a healthy oocyte for fertilisation and so have limited success in treating peri- or post-menopausal women without the use of donor eggs. Moreover, ARTs do little to tackle fundamental dysfunction within the ovary and in the oocytes that lead to female infertility and associated physiological adaptations.

The prospect of rejuvenating the exhausted ovary has been enticing ever since the description of oogonial stem cells (OSCs) in the ovarian cortex (White *et al.*, 2012), hinting at a possibility of therapeutic stimulation of post-natal folliculogenesis in subfertile women. In a study by Niikura *et al.* (2009), transplantation of ovarian stem cells from atrophic ovaries from aged mice into young, healthy counterparts resulted in their resumption of spontaneous oogenesis, suggesting that ovarian aging or insufficiency could be reversed if OSCs are provided a healthy environment. Further work has implicated a role for mitochondria in loss of oocyte quality associated with the aged ovary (Cozzolino et al., 2019); indeed, methods to replenish mitochondria within aged oocytes are currently being explored as a means to rejuvenate them (Labarta *et al.*, 2019). However, as with IVF, efforts to improve egg quality do not address the wider aspects of age-related ovarian dysfunction.

One recently proposed option for ovarian rejuvenation is the intraovarian injection of platelet-rich plasma (PRP) which is being used increasingly in clinical settings for a number of soft tissues, including to support wound healing and ligament and muscle repair (Suthar et al., 2017; Hurley *et al.*, 2019; Verma *et al.*, 2019; Zhang *et al.*, 2020). PRP was first described for ovarian rejuvenation by Pantos *et al.* (2016). Their work described how PRP, which is a component of blood, could, when in injected directly into the ovary, trigger the resumption of menstrual cycles in women exhibiting signs of the climacteric. In this review, we will briefly consider the concept of ovarian rejuvenation before describing what PRP is and how it is generated and finally reflecting on the current state of knowledge of ovarian rejuvenation with PRP.

## Ovarian rejuvenation

The paradigm that the mammalian ovarian reserve is fixed at birth dates back to a nineteenth century hypothesis by Waldeyer in 1870, which was reaffirmed by Zuckerman in 1951 (reviewed in Tilly *et al.*, 2009). However, there is mounting evidence that this is only part of the story, and that it may be possible to replenish the ovarian follicle pool due to the presence of a population of oogonial stem cells (OSC) in adult ovaries (Niikura *et al.*, 2009). It is likely that both of these explanations are true in part; there is a fixed number of follicles at birth, which declines until exhaustion (typically 40+ years of age in the human), but that a population of OSC co-exist in the ovary and may be activated under specific circumstances (Tilly and Telfer, 2009). However, spontaneous reactivation of OSCs is not yet believed to occur naturally *in vivo* in the adult human ovary. This is one principle that underpins the notion of ovarian rejuvenation.

As an illustration of this concept, mice rendered sterile from chemotherapeutic drugs can have fertility restored and can produce viable offspring through natural mating after undergoing an OSC transplant from neonatal or adult mouse ovaries (Zou *et al.*, 2009). It was further demonstrated that when ovarian tissue containing premeiotic germ cells from aged mice was transplanted into young host mice, the germ cells produced NOBOX-expressing oocytes and formed follicles (Niikura *et al.*, 2009). Combined, these studies show OSC transplantation may restore fertility and that it may be possible to produce oocytes from OSC from aged mammalian ovaries in the correct milieux.

Although data from animal models support the notion of OSCs, the presence of equivalent stem cell populations in humans remains disputed. For example, Virant-Klun *et al.* (2008) confirmed that ovarian stem-like cells were present on the surface epithelium of post-menopausal women and women with premature ovarian failure (POF), which aligns with the reported location of OSC in the ovaries of juvenile and young-adult mice (Tilly and Telfer, 2009). By contrast, when analysing the cell populations in the human ovarian cortex, Wagner *et al.* (2020) were unable to identify a population of germline stem cells. Of course, it must be acknowledged that studies on normal ovarian function in humans is rather constrained since substantial ovarian tissue from healthy, reproductive-aged women is rarely available. Furthermore, tissue from dysfunctional ovaries may not exhibit the full range of physiological function, and biopsies may not be reflective of the whole ovary as stem cells may not be uniformly spread (Horan and Williams, 2017). These factors make it challenging to determine definitively if a population of stem cells is present within the adult ovary.

If present, ovarian OSC may offer the potential for women experiencing ovarian failure as a result of menopause or POF to be treated for their infertility beyond the only current option of IVF using a donor egg. This has provided an underpinning of attempts to initiate ovarian rejuvenation in clinical settings, including investigating the utility of PRP in four pilot studies of different reproductive pathologies: POI, poor ovarian responders (POR), perimenopause and menopause (Sfakianoudis *et al.*, 2020b).

## Platelets and platelet-rich plasma

**Figure 1.**
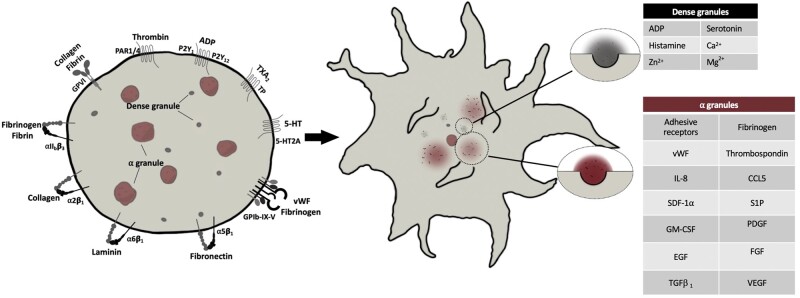
**Granule release in activated platelets.** Platelets express numerous glycoprotein, integrin and G- protein-coupled receptors that bind to a myriad of soluble and matrix proteins and molecules, resulting in tightly orchestrated intracellular signalling. This intracellular signalling significantly increases cytoplasmic calcium levels and causes drastic changes in the platelet cytoskeleton, resulting in ashape change in the platelet to an ‘echinocytic’ formation. During this process, granular storage compartments migrate inwards to the centre of the platelet and fuse with the plasma membrane and release their contents into the extracellular milleiu. PAR1/4, protease-activated receptors 1/4; GPVI, glycoprotein VI; TXA2, thromboxane A2; TP, thromboxane protstanoid receptor; 5-HT, 5-hydroxytryptomine; P2Y, purinergic receptor 2Y; vWF, von Willebrand Factor; IL-8, interleukin-8; CCL5, chemokine ligand 5; SDF-1a, stromal cell-derived factor 1 alpha; FGF, fibroblast growth factor; EGF, endothelial growth factor; GM-CSF, granulocyte-macrophage colony-stimulating factor; TGFb1, transforming growth factor beta 1.

The blood platelet is a tiny, anucleate cell responsible for the initiation of formation of a thrombus (Fig. 1). Platelets are formed from a fragment of megakaryocyte membrane that is pre-packaged with a myriad of molecules and complexes necessary for its primary function, which is to sense signs of trauma within the vasculature and aggregate together to stem the loss of blood. One of the primary steps in thrombus formation is platelet activation, which is driven by ‘outside-in’ signalling, initiated through a vast repertoire of G-protein coupled receptors, integrins and glycoprotein channels on the surface of the platelet (Li *et al.*, 2010). The activation of platelets can occur through numerous mechanisms by a seemingly endless number of agonists, including but not limited to, thrombin, collagen, adenosine diphosphate (ADP), thromboxanes, serotonin, oxidised LDL and extracellular divalent cations (Lopez-Vilchez *et al.*, 2009; Li *et al.*, 2010; Wraith *et al.*, 2013; Shen *et al.*, 2017).

A core platelet response to activation is the release of the contents of intracytoplasmic granules. Platelets contain two main granule stores, the alpha and dense granules, both of which replete with factors critical for an effective platelet response to vascular damage (Fig. 1). Where alpha and dense granules are lacking, the conditions grey platelet syndrome and delta storage pool deficiency can arise. Both of these conditions are associated with an increased bleeding tendency (Bolton-Maggs *et al.*, 2006). It is also of note that more recently, platelet secretory behaviour has been shown to extend beyond the realm of granular stores and also involves activation-dependent synthesis and release of cytokines and other bioactive molecules (Heijnen and van der Sluijs, 2015). It is, therefore, clear that the contents of platelet intracytoplasmic granules and *de* *novo* synthesis of agents are essential for the haemostatic response, and the descriptions on the functions of platelet releasate have historically focussed on its role in haemostasis (Rendu and Brohard-Bohn, 2001). However, the catalogue of bioactive proteins and molecules released by activated platelets can have multiple physiological effects which include increased angiogenesis, cell proliferation, cell differentiation and regulation or attenuation of apoptosis (Bir *et al.*, 2011; Au *et al.*, 2014; Golebiewska and Poole, 2015). The therapeutic role of the platelet releasate in driving tissue regeneration is of growing interest throughout modern medicine.

PRP is a term used to describe a fraction of the blood after processing. It is typically isolated from autologous whole blood retrieved by phlebotomy into a citrate-based anticoagulant. This is then subjected to differential centrifugation, resulting in the removal of red blood and immune cells, leaving behind a high concentration of platelets within plasma. Commercial sources of PRP are available, which can provide a predetermined concentration of platelets. However, in many cases, PRP is derived ‘in-house’, produced according to many subtle protocol variations. It is not uncommon for resulting PRP to retain varying concentrations of RBCs and WBCs; such contamination and absence of standardisation may result in conflicting findings regarding the effects of PRP in different applications.

In recent years, there has been significant interest in exploiting PRP in regenerative medicine. Particular attention has been paid to musculoskeletal (Scully *et al.*, 2019, 2020), oral-maxillofacial (Xu *et al.*, 2020) and osteoarthritis (Evans *et al.*, 2020) applications to name but a few. For a more comprehensive account, the reader is referred to a review (Scully *et al.*, 2018).

## PRP and ovarian rejuvenation: the evidence so far

Over the past decade, there have been a growing number of studies that have reported that injection of PRP directly into the ovary can increase folliculogenesis and egg harvest. One of the earliest studies reporting this approach was from Callejo *et al.* (2013), who implanted cryopreserved ovarian tissue within the peritoneum. PRP was used as a pro-angiogenic and proliferative agent, and the approach supported a successful live birth. The proangiogenic effect of PRP was further highlighted in a study by Bakacak *et al.* (2015), who used a rat model of ovarian ischaemia induced by torsion. In that study, PRP treatment in all conditions significantly increased peritoneal vascular endothelial growth factor (VEGF) and provided protection from ROS-induced oxidative damage during reperfusion.

More recently, direct injection of PRP into ovaries has been reported. In 2016, a short communication at the ESHRE Annual Meeting indicated that infusion of PRP into the ovary of perimenopausal women led to resumption of menstrual cycles (Pantos *et al.*, 2016). The study included only eight women but was the first reported use of PRP for rejuvenation of the perimenopausal ovary. Since then, there have been several limited investigations into the utility of PRP injection into the ovaries of perimenopausal women which are summarised in Table I. Sills *et al.* (2018) reported that for healthy women with a history of infertility, ovarian PRP infusion produced several MII oocytes for cryopreservation, with one individual proceeding to successful embryo transfer at time of publication. Other studies have reported similar cases; commonly ovarian PRP therapy has caused AMH to increase and FSH levels to fall in previous non-responders, leading to folliculogenesis, significant levels of oocyte retrieval, and in a handful of cases, spontaneous pregnancy (Sfakianoudis *et al.*, 2018; Farimani *et al.*, 2019; Pantos *et al.*, 2019; Hsu *et al.*, 2020).

**Table I:** **Summary of reports on the effect of PRP infusion in ovarian rejuvenation**.

Case reports		
Author	Findings summary	Procedure and controls
Sfakianoudis *et al.* (2018)	Spontaneous resumption of menstruation 6 weeks following PRP injection, with a concomitant reduction in FSH and increase in AMH being observed. A natural IVF cycle led to the retrieval of one high-grade oocyte that, after ICSI, resulted in a grade III 6-cell cleavage stage embryo. Following implantation, confirmation of a clinical pregnancy was determined, however the pregnancy spontaneously terminated at 5 weeks of gestation.	40-year-old woman with a history of premature menopause for 5 years and who was unable to naturally conceive for over a year. Approximately 4 ml of PRP (9 × 10^8^/ml) was injected into each ovary. Measurement of FSH, AMH and LH pre- and 6 weeks post-injection
Sfakianoudis *et al.* (2020b)	Menstruation was restored in the pilot study for POI patients (18 out of 30), AMH, FSH and AFC also significantly improved, noting 3 spontaneous pregnancies and live births. For the Poor Ovarian Responders (POR), there was an improvement to ICSI cycle performance. The perimenopausal pilot data showed 24 out of 30 women had improved hormone levels and AFC, as well as improved menstruation regularity, noting 4 spontaneous pregnancies and 3 live births. 13 out of 30 menopausal patients were described as positively responding to PRP treatment, noting 1 spontaneous pregnancy and live birth	Recruitment of a total 120 women suffering from POI, POR or who were perimenopausal or menopausal were assigned to 4 respective pilot studies. 4 ml of calcium gluconate-activated PRP (1 × 10^9^/ml) was injected into each ovary
Pantos *et al.* (2019)	Increased E_2_ and AMH and decreased FSH and LH were observed with PRP treatment. All participants resumed menstruation within 2 months post-injection and naturally conceived and carried until third trimester at time of publication	Injection of approximately 4 ml of activated PRP (concentration and agonist not reported) into the ovaries of three subfertile women who experienced >1 year of amenorrhea (2 POF, 1 menopausal). Measurement of FSH, AMH, E_2,_ LH and AFC pre- and post-therapy
Sills *et al.* (2018)	Multiple high-grade MII oocytes obtained from all participants, resulting in at least one Day 5 embryo per round of IVF, with one participant opting for immediate embryo transfer who then developed a clinical pregnancy. PRP injection was associated with a significant reduction in FSH	5 ml of PRP (concentration not reported) activated by calcium gluconate was injected throughout the ovaries of four women with at least one round of IVF failure or amenorrhoea for over 3 months. FSH, AMH and E_2_ measurements obtained pre- and post-PRP therapy. Hyperstimulation ovarian stimulation and oocyte retrieval performed from 59 days after therapy
Cakiroglu *et al.* (2020)	Spontaneous pregnancy was achieved in 23 out of 311 women diagnosed with POI, with 16 resulting in sustained implantation or livebirth. A significant increase in antral follicle count observed after PRP treatment, serum AMH increased after treatment, although serum FSH was not statistically significantly different. 201 patients developed antral follicles and attempted IVF (87 did not develop antral follicles), 57 of the 82 women who developed embryos underwent embryo transfer, 9 resulting in sustained implantation or livebirth	311 women aged 24–40 diagnosed with POI underwent intraovarian injection (in at least one ovary) of 2–4 ml of PRP (concentration not reported). PRP injection was timed randomly in amenorrheic women, and 10 days post-menstrual bleeding in oligomenorrheic women
Callejo *et al.* (2013)	PRP-loaded ovarian tissue that was implanted into the peritoneum on the broad ligament of a woman with no ovaries and was able to spontaneously resume menstruation. A round of IVF/ICSI on two obtained oocytes was able to provide generated two embryos for transfer, resulting in a clinical pregnancy. A healthy boy child was delivered by caesarean section at 38 weeks and 6 days, weighing 3.5 kg	Implantation of thawed cryopreserved ovarian tissue in a 30-year-old woman who had a bilateral oophorectomy at 20 years of age. Tissue was impregnated in a PRP gel and surgically implanted onto the broad ligament and growth factors administered. IVF/ICSI performed on resulting oocytes and implantation was performed with two Day 2 embryos
Farimani *et al.* (2019)	PRP-treatment increased the oocyte yield and the average number of retrieved oocytes and resulting embryos was higher after PRP treatment. 3 of the 12 women that underwent therapy had live births, two of which were via spontaneous conception and one with IVF	12 women suffering with poor ovarian reserve for more than 3 years underwent double ovarian stimulation and oocyte retrieval before and after injection of 2 ml of PRP (concentration not reported)
Hsu *et al.* (2020)	Resumption of folliculogenesis within 4 days post-PRP injection. Two rounds of hyperovulation supervovulation led to the capture of 6 oocytes, which after ICSI, led to two 8-cell and one 5-cell embryos, which were all transferred back into the uterus and resulted in a pregnancy of twins, which were delivered at 30 weeks with no documented abnormalities	33-year-old woman, with a history of irregular periods, who had several rounds of IUI cancelled due to lack of any follicles. Injection of approximately 4 ml PRP (concentration not reported) in conjunction with 1 ml 150 IU FSH and 75 IU LH throughout the ovarian tissue

**Table I:** **Continued**.

Basic research		
Author	Findings summary	Procedure and controls
Ahmadian *et al.* (2020)	VCD administration successfully reduced the presence of morphologically normal follicles to none and increased the atretic follicle count, also mildly increasing FSH levels although not significantly PRP intraovarian injection reduced follicular atresia in POI-induced rat ovaries and saw an increase in litter counts, as well as higher expression of *ANGPT2* and *KDR* when compared to the other groups. After PRP intervention, FSH levels declined, although not statistically significant, with the greatest decline observed in the higher platelet concentration of PRP	86 rats used, 63 were IP injected with 160 mg/kg VCD to induce POI, 18 received a similar volume of normal saline. 15 POI rats injected with 10 µl low concentrated PRP (8.5 × 10^5^/µl), 15 with 10 µl high concentrated PRP (21.6 × 10^5^/µl), 15 injected with 10 µl normal saline (sham), 15 without interference, 15 in control group (no POI and no PRP)
Bakacak *et al.* (2015)	PRP administration significantly reduced markers of reactive oxidant damage in and decreased histopathological damage scoring in the ovary when compared to sham injections. This response, however, was incomplete and remained significantly higher with PRP treatment compared to sham controls	Induction of ovarian torsion in rats. 60 female rats used. 12 used to prepare PRP, and 8 rats per group and 12 for PRP preparation: sham operation, ischemia, ischemia/reperfusion, sham operation + PRP, ischaemia+PRP, ischemia/reperfusion+PRP. Platelet concentration of PRP was 6.9 × 10^5^ ± 0.6 × 10^5^/µl was used
Cremonesi *et al.* (2020)	5 ml of PRP (1 × 10^9^ml) injected into the left right ovary of eight cows of proven fertility. PRP injection resulted in an increase in follicle count and increased subsequent number of grade 1–2 blastocysts	5 ml of PRP (1 × 10^9^/ml) was injected into the left ovary of Holstein–Friesian cows, leaving the right ovary as a pseudocontrol (no injection). Superovulation induced after the 9th day of cycle following PRP injection with Gn administration in decreasing doses for 5 days. Cows were then inseminated and oestrus was induced using PGF-2a. Embryo retrieval was then performed by flushing both left and right uterine horns
Hosseini *et al.* (2017)	An increase in follicle growth was observed in response to PRP (10%) supplementation. Interestingly, a mix of both PRP (5%) and FBS (5%) did not benefit follicle growth, suggesting a dose-dependent effect of PRP on follicle maturation	Primordial follicles were isolated from ovaries donated by three healthy women after death post-mortem. PRP (concentration not reported) was activated with 20 IU/ml thrombin. Follicles were embedded in a 3D gel matrix, supplemented with either 10% PRP, 5% PRP + 5% FCS, 10% FCS or 10% HSA with a-MEM media

**Non-randomised clinical trial**		
**Author**	**Findings summary**	**Procedure and controls**

Melo *et al.* (2020)	There were 11 clinical pregnancies, leading to 5 total live births in those receiving PRP injection, compared to 2 clinical pregnancies and 1 live birth in the untreated group. PRP therapy was associated with an increase in AMH and a decrease in FSH levels. Total AFC were higher post-therapy versus no intervention, with those seeking IVF/ICSI resulting in higher numbers of oocytes collected. Resulting embryos were graded higher in response to PRP when compared to no injection	Non-randomised interventional study (PRP vs. no injection) involving 83 women (46 PRP vs. 37 no injection). Each arm was then subdivided into those receiving IVF versus no IVF (timed conception and IUI). PRP (citrate anticoagulant, count not reported) was activated with 10% calcium chloride and injected as a 200 µl volume into each ovary

In the only preclinical study on the effect of PRP injection into human ovaries, Hosseini *et al.* (2017) obtained healthy donated ovaries from deceased donors. PRP injection led to an increase in follicle size and their viability at 10 days compared to treatment with foetal calf serum (FCS) alone. Surprisingly, a combination of FCS and PRP did induce follicular growth, which is an interesting observation worthy of further investigation.

While these case studies appear encouraging, it is important to reflect on the experimental designs. A common feature of the first studies of the effect of PRP infusion is the absence of a sham injection group. It is conceivable that the mechanical stretching and/or mild injury to the ovary resulting from the procedure is sufficient to elicit an inflammatory response leading to temporary resumption of ovarian function. For example, laparoscopic ovarian ‘drilling’ is a therapeutic option for the treatment of clomiphene-resistant PCOS (Lebbi *et al.*, 2015) and, thus, a comparable ovarian needle stick injury may be a causative factor in the success of PRP therapy. Importantly, the recent study of Ahmadian et al. (2020) used a sham injection group, which showed no morphologically normal follicles, and the same result was observed in the ‘no injection’ group. This demonstrated that injection with saline is not sufficient to reverse the effects of premature ovarian insufficiency in this animal model, nor can it elicit a comparable response to the two groups with different concentrations of PRP, which show reduced follicular atresia and increased follicular quality. It is vital that future studies control for this component of the intervention.

An important study was published by Melo et al. (2020) who reported findings from a non-randomised interventional study involving 83 subfertile women, 46 of whom opted for several infusions of 200 µl of autologous PRP into each ovary, and 37 who opted for no treatment. These two arms were further subdivided into groups who opted for IVF, and those who continued with unassisted conception. Overall, significantly higher antral follicle counts were observed in women who received PRP infusion compared to those women who received no treatment. In addition, embryo quality was scored higher from those obtained through PRP therapy, although there was no difference in the fertilisation rate of oocytes from either group. The authors concluded that ovarian injection of PRP did lead to increased egg yield in subfertile women and prompted changes within the oocyte which may lead to increased ‘quality’ of subsequent embryos. In both the IVF and spontaneous conception groups, those receiving PRP therapy developed 13 clinical pregnancies, compared to 2 in the control group although there were insufficient data on live births to draw any definitive conclusions. Although these data are encouraging, the absence of randomisation may have led to a socioeconomic selection bias, since PRP intervention was adopted only by couples able to pay for the treatment. Examples such as this illustrate the necessity that case studies are scrutinised in detail. Ideally, a properly controlled randomised clinical trial will be necessary to confirm the efficacy of ovarian PRP therapy.

## How might PRP induce ovarian rejuvenation?

Given the complexity of platelet signalling and activation, the precise details of how platelets initiate their full range of physiological effects remain unclear. However, it is well established that platelets release a range of cytokines in response to activation (Roh *et al.*, 2016). Cytokine signalling is increasingly being shown to be involved in the interrelationship among the oocyte, granulosa and thecal cells, with dysfunction in this ecosystem resulting in deficiencies in follicle maturation, ovulation and luteinisation (Orisaka *et al.*, 2006; Field *et al.*, 2014). A number of the cytokines that regulate follicle development are released by platelets through secretion of their alpha and dense granule contents during platelet activation (Table II). Therefore, a working hypothesis is that PRP may provide a readily accessible, individualised, cost-effective blend of proangiogenic, proliferative and proinflammatory factors which may stimulate de-novo oogenesis and/or follicle maturation.

**Table II:** **Factors released by platelets with known effects in the ovary**.

Factor	Effect	Plasma	Platelet	References
**BMPs**	Essential for oocyte maturation and folliculogenesis. Involved in maintaining cumulus cell expansion. BMP2 expression associated with an increase in oocyte quality scoring	✓	✓	Hussein *et al.* (2005) Kalén *et al.* (2008) Demiray *et al.* (2017)
**CCL5**	Higher CCL5 levels in follicular fluid associated with increased subsequent embryo quality upon IVF	✓	✓	Ledee *et al.* (2008) Machlus *et al.* (2016)
**EGF**	Required for LH-mediated cumulus cell expansion	✓	✓	Ben-Ezra *et al.* (1990) Reizel *et al.* (2010)
**IL-8**	Associated with higher pregnancy rates and embryo quality. Found in healthy follicular fluid	✓	✓	Arici (1996) Huang *et al.* (2017)
**PDGF**	Expression of PDGF receptors in oocytes and granulosa cells. Inhibition of PDGFR in rat ovaries results in increased follicle atresia, reduction in primary/early and antral follicle formation and intraovarian blood vessel size. Shown to increase the stromal cell migration from the fallopian tube fimbriae towards the ovulating follicle. Involved in primordial to primary follicle transition	✓	✓✓✓	Hart *et al.* (1990) Nilsson *et al.* (2006) Valeri *et al.* (2006) Pinkas *et al.* (2008) Pascuali *et al.* (2015) Yeh *et al.* (2016)
**PF4/CXCL4**	Strong chemoattractant for neutrophils and monocytes, inducing robust phenotypic alterations. Increased intrafollicular levels of PF4 found in those with PCOS	✓	✓✓✓	Deuel *et al.* (1981) Pervushina *et al.* (2004) Huang *et al.* (2016)
**P-selectin (CD62)**	PSGL-1 expression in the porcine zona pellucida. Key in the recruitment of neutrophils to sites of injury	?	✓✓✓	Geng *et al.* (1997) Merten and Thiagarajan (2000)
**SDF-1α/CXCL12**	Causes inhibition of primordial to primary follicle transition in murine neonates, resulting in smaller, more dense yet numerous oocytes. Associated with a higher preovulatory follicle size in humans. Encourages the migration of T-cells, increases granulosa cell survival and overall oocyte quality	✓	✓	Kryczek *et al.* (2005) Holt *et al.* (2006) Massberg *et al.* (2006) Nishigaki *et al.* (2011)
**Serotonin**	5-HT receptors robustly expressed in human ovarian epithelium. Both Serotonin and 5-HT transporters expressed in murine cumulus-oocyte complexes. Tryptophan hydroxylase robustly expressed in cumulus cells. Shown to modulate oestradiol production in cultured rat follicles	✓	✓✓✓	Amireault and Dubé (2005) Brenner *et al.* (2007) Henriksen *et al.* (2012) Cloutier *et al.* (2018)
**TGF-β1**	Strongly regulates follicle survival and apoptosis. Synergises with VEGF to regulate angiogenesis. Essential in the crosstalk between thecal cells, granulosa cells and the oocyte during folliculogenesis and maturation. Critical for transcriptional activity through Smads	✓	✓✓	Assoian *et al.* (1983) Dragovic *et al.* (2007) Meyer *et al.* (2012)
**TSP-1**	Present in granulosa cells, follicle antra and stromal compartment. Increases migration of ovarian vascular endothelial cells in primates. Inhibition of thrombospondin diminishes follicle rupture and oocyte release. CD36 observed in both murine and human oocytes and shown to co-determine fertilisation rate with BAI1/3	✓	✓✓✓	Jaffe *et al.* (1982) Disdier *et al.* (1989) Kõks *et al.* (2009) Bender *et al.* (2019) Rival *et al.* (2019) Zaslavsky *et al.* (2010)
**VEGF**	Regulates intraovarian vascular events, leading to increased oxygen and nutrient supply. Causes increased follicle growth and corpus luteum formation and function	✓	✓✓	Shweiki *et al.* (1993) Wynendaele *et al.* (1999) Duncan *et al.* (2008) Italiano *et al.* (2008)
**TIMP-4**	Complexed with MMP-2 within the cytoplasm of platelets. Platelet activation causes dissociation of the complex and efflux out of the platelet. TIMP-4 is widely expressed in the murine ovary and has been shown to regulate morphogenesis and corpus luteum longevity during pregnancy	✓	✓✓	Radomski *et al.* (2002) Bu *et al.* (2006)
**GM-CSF**	Present in low levels in platelets and shown to prevent eosinophil apoptosis. High expression of α and β GM-CSF receptors in cumulus cells	✓	✓	Raidem *et al.* (2003) Lee *et al.* (2008) Peralta *et al.* (2013)
**FGF**	Drives folliculogenesis and follicle maturation, Increases proliferation of thecal, granulosa and stromal cells. Expression of multiple FGF receptor isoforms in oocytes and granulosa cells	✓	✓✓	Nilsson *et al.* (2001) Pintucci *et al.* (2002) Ben-Haroush *et al.* (2005)
**S1P**	Abundant in follicular fluid aspirates. May increase folliculogenesis through activation of HIPPO signalling, leading to increased *CCN2* expression	✓	✓✓✓	Ono *et al.* (2013) Cheng *et al.* (2015) Urtz *et al.* (2015) Pors *et al.* (2020)

BMP, bone morphogenic protein; CCL5/RANTES, chemokine (C-C motif) ligand 5; EGF, endothelial growth factor; IL-8, interleukin-8; PDGF, platelet-derived growth factor; PF4, platelet factor 4; CXCL4, chemokine (C-X-C motif) ligand 4; SDF-1a, stromal-cell derived factor 1 alpha; CXCL12, chemokine (C-X-C motif) ligand 12; TGF-b1, transforming growth factor beta 1; VEGF, vascular endothelial growth factor; TIMP-4, tissue inhibitor of matrix metalloprotease; TSP-1, thrombospondin-1; GM-CSF, granulocyte-monocyte colony stimulating factor; FGF, fibroblast growth factor; S1P, sphingosine-1-phosphate; *CCN2*, connective tissue growth factor.

One possible explanation of the observed effects of PRP on the ovary might be that it acts in a proangiogenic manner (Kakudo *et al.*, 2014) via action of platelet-released cytokines (Table II), including, for example VEGF. Primordial follicles typically rely on stromal blood vessels, but become progressively encapsulated in a thecal capillary network during maturation, a process which is mirrored by increased VEGF expression that persists through to corpus luteum formation (Gordon *et al.*, 1996; Barboni *et al.*, 2000; Danforth *et al.*, 2003; Pauli *et al.*, 2005). Heterozygous knockdown of the hypoxia-response element within the VEGFA promoter or VEGFR antagonism in mouse ovaries leads to vascular malformation, resulting in a poor ovarian response to stimulation (Feng *et al.*, 2017), indicative of a role for VEGF in follicle development and the overall importance of correctly regulated vascularisation in follicle development. Another major constituent of platelet releasate, platelet-derived growth factor (PDGF), has also been implicated in regulating vessel formation and maturity. This was demonstrated via intraovarian injection with an anti-PDGF antibody in rats by Pascuali *et al.* (2015), who consequently observed a reduction in follicle maturation paired with an increase in follicle atresia. This direct evidence for the importance of proangiogenic factors in follicle development supports the idea that PRP and/or platelet releasate can increase blood supply to the immature follicle pool and/or OSCs and encourage their maturation.

An additional potential explanation for the positive effects of PRP on the ovary is via sphingosine-1-phosphate (S1P) (Ono *et al.*, 2013; Urtz *et al.*, 2015). S1P has been isolated from follicular fluid at high nanomolar concentrations (Von Otte *et al.*, 2006) and there is evidence to suggest that it can promote follicle maturation, likely through increased expression of CCN2, a connective tissue growth factor shown to drive follicle maturation (Cheng *et al.*, 2015). Platelet alpha granules contain abundant stores of S1P, which is released upon activation and have been measured at over 300 nM per 1 × 10^7^ platelets. If a linear relationship between S1P concentrations and platelet count exists, this would estimate that in studies that have infused activated PRP into the ovary, the amount of S1P delivered is approximately 9 µM, close to the range reported to be beneficial by Cheng *et al.* (2015). However, a recent study involving both murine and human follicles and human-to-murine xenotransplantation reported that although *CCN2* expression was elevated in response to supraphysiological S1P doses, there was no increase in the number of follicles. By contrast, ovaries receiving S1P treatment suffered a significant reduction in follicle number compared to control counterparts (Pors *et al.*, 2020). These findings again highlight the uncertainty of the effect of factors released by activated platelets on oocyte and follicle development and clinicians must be careful when considering such approaches.

Although it is theorised that PRP supports the development of follicles from OSCs, alternative explanations must be considered. In a study by Hosseini *et al.* (2017), PRP was found to improve the growth and viability *in vitro* of preantral follicles isolated from human ovaries post-mortem, supporting the notion that PRP may aid ovarian rejuvenation through supporting development of existing primordial follicles. However, this application relies on the patient having a supply of oocyte-containing follicles, thus, rendering the approach unsuitable for women who have experienced ovarian exhaustion. Panda *et al.* (2020) expressed the need for better-controlled studies to confirm the conclusions drawn by Cakiroglu *et al.* (2020), which found that the number of remaining follicles within the ovaries of women with POI determines their response to PRP infusion, and that women without any antral follicles are unlikely to respond to PRP.

The prospective pilot study by Sfakianoudis *et al.* (2020b) determined that perimenopausal women and women deemed to be POR benefitted the most from the treatment, more so than POI and menopausal patients. In an article by Sfakianoudis *et al.* (2020a), they describe how novel techniques (such as PRP, ovarian stem cells transplant and ovarian tissue transplant) may effectively treat ovarian insufficiency by reactivating follicular growth through restoring the microenvironment of the ovary. Therefore, it should be acknowledged that PRP infusion may only be an appropriate treatment for select ovarian disorders.

## PRP: a note of caution

A primary consideration of the effect of PRP in any aspect of regenerative medicine is the ‘activation status’ of the platelet (Fig. 1). As previously discussed, platelets have the capacity to respond to agonists and release a range of molecules, creating a ‘releasate’ (Piersma *et al.*, 2009; Parsons *et al.*, 2018). Indeed, PRP from resting platelets differs markedly to that containing activated platelets, and the mode of activation will influence the composition of the releasate. Despite this, there is considerable variation in the activation status of platelets used in studies of ovarian rejuvenation; some studies describe using calcium (Sills *et al.*, 2018; Hsu *et al.*, 2020; Melo et al., 2020) or thrombin (Hosseini *et al.*, 2017), while others inject quiescent platelets or simply do not state their activation status (Callejo *et al.*, 2013; Farimani *et al.*, 2019; Pantos *et al.*, 2019). The importance of reporting the activation status and the methods therein, paired with the use of appropriate controls, is critical, given reported effects of thrombin or calcium alone in the regulation of ovarian function. For example, thrombin has been shown to regulate progesterone synthesis in the preovulatory ovary homogenates, with multiple cell types within the ovary readily expressing PAR1 and PAR4 receptors (Cheng *et al.*, 2012) through which thrombin elicits biological function directly. In addition, there is good evidence of an interaction between calcium signalling and ovarian steroidogenesis (reviewed in Kouba *et al.*, 2019).

To date, it appears that efforts to investigate the role of platelet activation in the context of ovarian rejuvenation remain limited. For example, platelets possess CD40 and αIIbβ_3_ on their surface in a resting state (Inwald *et al.*, 2003; Li *et al.*, 2010). Thus, it is conceivable that these adhesive receptors and ligands are sufficient to elicit folliculogenesis or to recruit immune cells to the ovary without the need for platelets within the PRP to have become activated prior to injection. By contrast, activation and subsequent degranulation may be the critical function required for PRP to elicit an effect and quiescent PRP may become activated through exposure to platelet-activating matrices within the ovarian stroma. Differentiating the effects of stimulated versus unstimulated PRP should be a focus of future investigations and may help isolate the most effective agents that cause the reported regenerative effect in the ovary, paving the way for more defined interventions.

The contents of platelet granules may not all be beneficial for re-establishing female fertility among all disease settings. As a theoretical example, thrombospondin-1 has been implicated in follicle development (Kõks *et al.*, 2010; Bender *et al.*, 2019), yet it inhibits the proangiogenic action of VEGF (Greenaway *et al.*, 2007) which may be undesirable where perfusion of the ovaries is limited. In addition, increased intraovarian VEGF and blood flow is thought to play a role in the pathogenesis of PCOS (Chan *et al.*, 2003; Carmina *et al.*, 2005; Peitsidis and Agrawal, 2010). Conversely, Anvari *et al.* (2019) recently reported that PRP therapy partially re-established hormonal balance in a rat model of PCOS. Here, PRP treatment increased the expression of oestrogen receptors α and β and of superoxide dismutase and glutathione peroxidase in ovarian homogenates. PRP-treated ovaries had significantly more pre-antral and antral follicles up to 30 days after treatment, suggesting that PRP may be a viable option for driving folliculogenesis in females with PCOS. In addition, platelets also release significant quantities of IL-15 when activated (de Miguel-Gómez et al., 2020). Increased IL-15 concentrations in follicular fluid have been negatively correlated with pregnancy outcomes via IVF, indicating that this cytokine may be detrimental to follicle maturation (Spanou *et al.*, 2018). Interestingly, it is highly expressed in immature follicles, and falls during their maturation, which raises the potential importance of IL-15 in the activation of germline stem cells, as IL-15 is a potent regulator of other stem cell types (Huntington *et al.*, 2009; Gómez-Nicola *et al.*, 2011). This interplay and opposing effects of PRP constituents in different contexts serve to illustrate the importance of detailed studies of the mechanisms of how PRP might act on the ovary, and much additional work is required before any conclusions can safely be drawn.

## Conclusions and future prospects

Even though there are other biological derivatives, such as human umbilical cord plasma, which may provide additional benefits in the sphere of ART (de Miguel-Gómez et al., 2020), the appeal of PRP lies in its balance among therapeutic effect, cost effectiveness, ease of isolation and autologous nature. However, with the increased interest of ovarian PRP injection inconclusive efficacy and lack of understanding of mechanism of action, fundamental research into the effect of this therapy on the cellular level is required. Dysregulation of early processes in oocyte maturation and subsequent embryo development can lead to drastic changes in the growing foetus, possibly leading to increased risk of disease in early years and onwards. Indeed, the long-term safety of new treatments in ART must be robustly assessed (Harper *et al.*, 2012), especially given the context that there is still concern that ART itself may increase the risk of birth defects (Luke *et al.*, 2020). Perhaps most of most relevance in the context of PRP, the precise physiological causes of premature ovarian failure or primary ovarian insufficiency outside the natural ageing process remain poorly understood, and further work to understand the role of putative OSCs is required.

With ovarian PRP therapy in its infancy, understandably, there is poor standardisation among research groups and clinics, however, this must soon be addressed to form a consensus as to the efficacy of this treatment. To assist this, we would propose that authors carrying out research in this area commit to reporting of key basic information regarding PRP. At a minimum, we suggest that such studies should include platelet count, activation status, activation agent (if any), platelet function testing, origin of PRP, volume infused, anticoagulant used, clinical account of menstrual status based on AMH level, and a detailed reporting of the participant’s fertility history.

Additionally, while there are encouraging data supporting the notion that PRP treatment might have some future use in the ART setting, it is paramount that we undertake robust and detailed basic studies to understand mechanism of action and to try to identify unintended outcomes, before moving into whole animal studies. These precursors would be an important bedrock on which to carry out well-designed clinical studies, allowing us to investigate this new technology with all rigour currently available. There are, at the time of writing, 13 registered clinical trials investigating the effect of PRP on ovarian rejuvenation either recruiting or underway. Strikingly, few of these trials describe the inclusion of appropriate PRP controls. It is only from well-controlled trials, built on detailed mechanistic understanding that we can clarify the platelet-mediated effects of PRP therapy in ovarian rejuvenation and folliculogenesis.

## Data availability

No new data were generated or analysed in support of this research.
